# Schizophrenia as a risk factor for cardiovascular and metabolic health outcomes: a comparative risk assessment

**DOI:** 10.1017/S2045796023000045

**Published:** 2023-02-09

**Authors:** S. Ali, D. Santomauro, A. J Ferrari, F. Charlson

**Affiliations:** 1 School of Public Health, The University of Queensland, Brisbane, Australia; 2 Queensland Centre for Mental Health Research, Brisbane, Australia

**Keywords:** Epidemiology, health outcomes, mental health, schizophrenia

## Abstract

**Aims:**

Cardiometabolic diseases are responsible for the majority of premature deaths in people with schizophrenia. This study aimed to quantify the fatal burden of ischaemic heart disease (IHD), stroke and diabetes attributable to schizophrenia.

**Methods:**

Comparative Risk Assessment methodology from the Global Burden of Disease (GBD) study was used to calculate attributable burden; pooled relative risks (RRs) for IHD, stroke and diabetes were estimated via meta-regression, which were combined with GBD schizophrenia prevalence estimates to calculate the deaths and years of life lost (YLLs) caused by these health outcomes that were attributable to schizophrenia. The proportion of explained all-cause fatal burden and corresponding unexplained burden was also calculated.

**Results:**

The pooled RRs for IHD, stroke and diabetes mortality were 2.36 [95% uncertainty interval (UI) 1.77 to 3.14], 1.86 (95% UI 1.36 to 2.54) and 4.08 (95% UI 3.80 to 4.38) respectively. Schizophrenia was responsible for around 50 000 deaths and almost 1.5 million YLLs globally in 2019 from these health outcomes combined. IHD, stroke and diabetes together explained around 13% of all deaths and almost 11% of all YLLs attributable to schizophrenia, resulting in 320 660 (95% UI 288 299 to 356 517) unexplained deaths and 12 258 690 (95% UI 10 925 426 to 13 713 646) unexplained YLLs.

**Conclusions:**

Quantifying the physical disease burden attributable to schizophrenia provides a means of capturing the substantial excess mortality associated with this disorder within the GBD framework, contributing to an important evidence base for healthcare planning and practice.

## Introduction

People with schizophrenia have a decreased life expectancy of 13 to 15 years (Hjorthøj *et al*., 2017). While this population experiences higher rates of deaths from unnatural causes compared to the general population, most premature deaths are attributable to natural causes (Lawrence *et al*., 2010). The most common cause of death among people with schizophrenia in high-income countries (HICs) is cardiovascular disease (CVD), accounting for approximately one-quarter of male deaths and one-third of female deaths, with limited data available from low- and middle-income countries (LMICs) (Lawrence *et al*., 2013; Olfson *et al*., 2015; Westman *et al*., 2018; Laursen *et al*., 2019; Pan *et al*., 2020; Ali *et al*., 2022). The leading cause of CVD deaths worldwide are ischaemic heart disease (IHD) and stroke, although mortality rates have declined dramatically in HICs over the past 50 years due to a reduction in risk factors and improved medical care (Lopez and Adair, 2019; GBD Collaborative Network, 2020b). However, people with schizophrenia have not benefitted from these improvements and experience higher mortality following CVD diagnoses compared to people without schizophrenia (Kugathasan *et al*., 2018; Yung *et al*., 2019). Diabetes mellitus is an established risk factor for CVD and associated with greater severity and higher fatality (The Emerging Risk Factors Collaboration, 2010; Leon and Maddox, 2015; Zheng *et al*., 2018). Diabetes is highly prevalent in people with schizophrenia, affecting around 1 in 10 people, with elevated diabetes-related mortality compared to people with diabetes only (Vancampfort *et al*., 2016; Toender *et al*., 2020). The pervasive mortality gap experienced by people with schizophrenia in regard to these preventable and manageable cardiometabolic diseases is not currently reflected in global health estimates, including the Global Burden of Disease (GBD) study.

GBD measures the disability and death caused by diseases, injuries and risk factors, which is critical for informed policy-making and shaping health systems to meet the needs of the populations they serve. Mortality, or fatal burden, is not only measured through number of deaths but also years of life lost (YLLs), which is calculated by subtracting the age at death from the longest possible life expectancy for a person at that age. GBD adheres to the International Classification of Diseases (ICD-10) death-coding system, which attributes death to a single underlying cause; mental disorders are rarely listed as the underlying cause of death on death certificates and premature deaths are captured under other causes (Whiteford *et al*., 2013; Vigo *et al*., 2016). For example, the death of someone with schizophrenia who dies from IHD will be attributed entirely to IHD, regardless of the contribution of schizophrenia to the premature death. Subsequently, there are very few deaths attributed to mental disorders in GBD (GBD 2019 Mental Disorders Collaborators, 2022). GBD's Comparative Risk Assessment (CRA) methodology offers a means of investigating the contribution of other underlying causes of death while circumventing death coding practices (GBD 2019 Risk Factors Collaborators, 2020). This methodology is used to quantify and compare the contribution of risk factors to disease burden by estimating attributable burden – the difference between the burden currently observed and the burden that would have been observed under a counterfactual level of risk factor exposure (Ezzati *et al*., 2002). Framing schizophrenia as a risk factor for other health outcomes, such as CVD and diabetes, allows for the contribution of the mental disorder to the burden of these diseases to be quantified.

Unlike more proximal risk factors where reducing the distribution of the risk factor itself will improve population health, the disease burden attributable to schizophrenia can be prevented by addressing modifiable factors in health-related behaviours and health services (Firth *et al*., 2019). These estimates can therefore play an important role in healthcare policy and service planning, providing evidence to integrate agendas on mental health and non-communicable diseases, as well as for coordinated care and primary prevention. We will also be examining how much of the overall fatal burden attributable to schizophrenia (i.e., all-cause mortality) is accounted for by cardiometabolic diseases, in order to quantify how much burden remains to be explained.

This study aims to estimate the mortality risk of IHD, stroke and diabetes in people with schizophrenia and quantify the fatal burden of these physical health outcomes attributable to schizophrenia, as well as the proportion of explained all-cause burden and corresponding unexplained burden.

## Methods

### Overview

We used CRA methodology to estimate the burden attributable to schizophrenia as a risk factor for IHD, stroke and diabetes (GBD 2019 Risk Factors Collaborators, 2020). This process consisted of five key steps:

1. Establishing that there is sufficient evidence for causal relationships between the risk factor and outcomes; a number of comprehensive review articles have been published outlining the mechanisms, including biological pathways, linking schizophrenia to CVD (Ringen *et al*., 2014; Nielsen *et al*., 2020; Lemogne *et al*., 2021), and diabetes (Ward and Druss, 2015; Mamakou *et al*., 2018; Mizuki *et al*., 2020).
2. Estimating the relative risk (RR) of each outcome due to the risk factor; we compiled RRs of IHD, stroke and diabetes mortality for persons with schizophrenia and pooled these estimates using meta-regression.
3. Estimating exposure levels of the risk factor; we compiled prevalence estimates of schizophrenia from the GBD 2019 study.
4. Determining the counterfactual level of exposure, known as the theoretical minimum risk exposure level (the distribution of a risk that would lead to the greatest improvement in population health); we defined this as the absence of schizophrenia within the population.
5. Calculating population attributable fractions (PAFs) and attributable burden: we combined the pooled RR estimates with the prevalence estimates to generate PAFs, which were then multiplied by the underlying fatal burden (deaths and YLLs) of each health outcome to estimate attributable burden.

Additionally, the RR of all-cause mortality for schizophrenia was used to calculate the proportion of explained fatal burden and corresponding unexplained burden attributable to schizophrenia.

### Case definitions

Schizophrenia was defined according to ICD or the Diagnostic and Statistical Manual of Mental Disorders (DSM) diagnostic criteria: ICD-10 code F20 and DSM-IV code 295.90, to align with the GBD prevalence data. However, we also explored the utility of including studies with a wider case definition that included schizoaffective disorder (ICD-10 code F25, DSM-IV code 295.70) via a covariate in the meta-regression detailed below due to its very low prevalence and likely minimal impact on the overall estimates (Laursen *et al*., 2007).

The health outcomes were also defined according to ICD coding; IHD (which is used interchangeably with coronary heart disease): ICD-10 codes I20–25 or ICD-9 codes 410–414; stroke: ICD-10 codes I60–69 or ICD-9 codes 430–438; and diabetes: ICD-10 codes E10–E14 or ICD-9 code 250.

### Prevalence of exposure

Prevalence data for schizophrenia was obtained from GBD 2019, with detailed methods available elsewhere (GBD 2019 Mental Disorders Collaborators, 2022). Briefly, these estimates are based on a systematic literature review, which included surveys with representative samples of the general population reporting past-year schizophrenia prevalence or less. DisMod-MR 2.1, a Bayesian meta-regression tool, was used to produce pooled prevalence estimates by age and sex for 204 countries and territories. Global age- and sex-specific data for 2019 was used in this study. All GBD 2019 analyses complied with the Guidelines for Accurate and Transparent Health Estimates Reporting statement (GBD 2019 Diseases and Injuries Collaborators, 2020; GBD 2019 Mental Disorders Collaborators, 2022).

### Relative risk estimates

Studies containing estimates of IHD, stroke and diabetes mortality in people with schizophrenia were identified from a previous systematic review detailed elsewhere (Ali *et al*., 2022), which adhered to the Preferred Reporting Items for Systematic Reviews and Meta-Analyses (PRISMA) 2020 statement. In brief, the online databases PubMed, EMBASE and PsycINFO were searched from 1/1/1980 to 31/12/20 for studies examining excess mortality in people with severe mental disorders (SMD). Studies were eligible if they were longitudinal; the study population was diagnosed according to established criteria, not restricted to subgroups and the disorder was primary and not acute or transient; and mortality was reported in comparison to the general population or a control group without SMD. Details of the data extraction process are provided in the online Supplementary material (page 1). The following effect measures were included and treated as equivalent measures of mortality risk; standardised mortality ratio (SMR), hazard ratio (HR) and relative risk (RR) (including mortality rate ratios); HRs and SMRs were converted to RRs where possible as detailed in (Shor *et al*., 2017) and (Jones and Swerdlow, 1998) respectively. Risk of bias was assessed using an adaptation of the Newcastle-Ottawa scale (Wells *et al*., 2019). A summary of the included studies can be found in Table 1.

**Table 1.** Summary of included studies

Study	Country	Diagnoses	Sample size	Observation period	Population type	Outcomes
Castagnini *et al*. (2013)	Denmark	SZ	4576	1995–2008	Inpatient and outpatient	Stroke
Crump *et al*. (2013)	Sweden	SZ	8277	2003–2009	Inpatient and outpatient	IHD, stroke, diabetes
Ko *et al*. (2018)	Taiwan	SZ	4298	1998–2010	Inpatient	Stroke, diabetes
Lahti *et al*. (2012)	Finland	SZ + SA	204	1969–2003	Inpatient	IHD, stroke
Laursen *et al*. (2007)	Denmark	SZ	Not reported	1973–2001	Inpatient	Stroke
Laursen *et al*. (2013)	Denmark, Finland, Sweden	SZ	20430, 20835, 24823	2000–2007	Inpatient	IHD, stroke
Laursen *et al*. (2019)	Denmark	SZ	47 554	1995–2015	Inpatient and outpatient	Diabetes
Lawrence *et al*. (2003)	Australia	SZ	Not reported	1980–1998	Inpatient and outpatient	IHD
Olfson *et al*. (2015)	USA	SZ + SA	1 138 853	2001–2007	Inpatient and outpatient	IHD, stroke, diabetes
Osborn *et al*. (2007)	UK	SZ	Not reported	1987–2002	Outpatient	IHD, stroke
Osby *et al*. (2000)	Sweden	SZ	7784	1973–1995	Inpatient	Stroke
Pan *et al*. (2020)	Taiwan	SZ + SA	95632, 104561	2005–2008, 2010–2013	Inpatient and outpatient	Diabetes
Westman *et al*. (2018)	Sweden	SZ + SA	46 911	1987–2010	Inpatient	IHD, stroke
Yung *et al*. (2021)	China	SZ + SA	46 896	2006–2016	Inpatient and outpatient	IHD, stroke

SZ, schizophrenia; SA, schizoaffective disorder; IHD, ischaemic heart disease.

Multi-level meta-regression (with estimates nested within each study) was used to pool mortality estimates for each health outcome separately using the *metafor* package (Viechtbauer, 2010) in R (version 4.1.2). The following covariates were tested as potential sources of heterogeneity as guided by the findings of Ali *et al*., 2022: population type (inpatient and outpatient combined vs inpatient only), case definition (schizophrenia vs schizophrenia and schizoaffective disorder combined), sex, age and age-sex interaction. Backward elimination using the Akaike information criterion was used to develop the final model for each health outcome. Further details of the analysis methods are provided in the online Supplementary material (page 1). In terms of all-cause mortality, the meta-regression model for schizophrenia in Ali *et al*., 2022 (see page 1 of the online Supplementary material for details on covariates) was used to derive age- and sex-specific RRs for the calculation of unexplained burden described below; as a summary estimate, the adjusted RR for both sexes was 2.89 (95% UI 2.50 to 3.34) based on 23 studies and 70 estimates.

### Attributable burden

Using R, PAFs were calculated by age and sex using the following formula (GBD 2019 Risk Factors Collaborators, 2020):
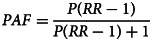
Where *P* is the global prevalence of schizophrenia and RR is the adjusted relative risk of each health outcome. Age-sex-specific PAFs were multiplied by the corresponding GBD 2019 deaths and YLLs for each health outcome (GBD 2019 Diseases and Injuries Collaborators, 2020) to calculate age-sex-specific attributable burden. All-age and both-sex data was population weighted using GBD 2019 population estimates (GBD Collaborative Network, 2020a). The proportion of deaths and YLLs attributable to schizophrenia was calculated by dividing the attributable burden by the total burden for each health outcome. To estimate the unexplained fatal burden and proportion of explained fatal burden for schizophrenia, attributable burden was also calculated for all causes. Unexplained burden was calculated by subtracting the attributable burden for each health outcome from the all-cause attributable burden. A Markov chain Monte Carlo simulation was conducted and 1000 samples from the probability distributions of the RRs, prevalence, deaths, YLLs and population estimates were pulled in order to propagate all of these sources of uncertainty into the final estimates. Prevalence and deaths were logit-transformed to ensure all samples remained between 0 and 1, and YLLs were log-transformed to ensure all samples were above 0. The reported estimates and 95% uncertainty intervals (UI) correspond to the mean and 2.5th and 97.5th quantiles of the samples.

## Results

### Pooled relative risks

A total of 14 studies covering 8 countries were included in the analyses for IHD, stroke and diabetes mortality (see pages 2–3 of the online Supplementary material for the results of the search and selection process). The final meta-regression model coefficients and pooled RRs can be found in Table 2. For IHD, 8 studies provided 49 estimates resulting in an RR of 2.36 (95% UI 1.77 to 3.14) for both sexes. Sex, age, age-sex interaction and case definition were included in the final model, the latter of which was not significant. The RR for stroke was 1.86 (95% UI 1.36 to 2.54) for both sexes based on 11 studies and 56 estimates, with significant age effects. Population type was also included as a covariate in the final model but was not statistically significant. Diabetes had the largest RR, 4.08 (95% UI 3.80 to 4.38), from 5 studies and 20 estimates, with significant effects of sex, age and population type (inpatients only had a larger RR compared to inpatients and outpatients). RRs were larger in females for IHD: 2.73 (95% UI 2.04 to 3.64) compared to 2.04 (95% UI 1.53 to 2.72) for males; and diabetes: 4.84 (95% UI 4.48 to 5.24) compared to 3.43 (95% UI 3.17 to 3.72). RRs decreased with age for all three health outcomes.

**Table 2.** Final meta-regression model coefficients and relative risks by health outcome

Covariate	*β* (95% UI)	*p*	RR (95% UI)
IHD (*n* = 8 studies, 49 estimates^a^)			
Intercept	0.86 (0.57–1.15)	<0.001	2.36 (1.77–3.14)
Sex	0.29 (0.25–0.33)	<0.001	
Age	−0.02 (−0.02 to −0.02)	<0.001	
Age-sex	−0.01 (−0.02 to −0.01)	<0.001	
Case definition			
SZ (*n* = 5)	Reference		
SZ + SA (*n* = 4)	0.35 (−0.09 to 0.79)	0.116	
Stroke (*n* = 11 studies, 56 estimates^a^)			
Intercept	0.62 (0.31–0.93)	<0.001	1.86 (1.36–2.54)
Age	−0.01 (−0.01 to <−0.01)	<0.001	
Age-sex	<−0.01 (−0.01 to <−0.01)	0.039	
Population type			
Inpatient + outpatient (*n* = 4)	Reference		
Inpatient (*n* = 7)	0.32 (−0.09 to 0.73)	0.124	
Diabetes (*n* = 5 studies, 20 estimates^a^)			
Intercept	1.41 (1.33–1.48)	<0.001	4.08 (3.80–4.38)
Sex	0.34 (0.28–0.41)	<0.001	
Age	−0.01 (−0.02 to −0.01)	<0.001	
Population type			
Inpatient + outpatient (*n* = 4)	Reference		
Inpatient (*n* = 1)	0.97 (0.41–1.52)	<0.001	

UI, uncertainty interval; RR, relative risk; IHD, ischaemic heart disease; SZ, schizophrenia; SA, schizoaffective disorder. Note: RRs are for both sexes and correspond to the exponentiated intercept of each multivariate meta-regression model; sex covariate corresponds to per cent female and age corresponds to mid-point of age-range centred at mean age.

a Each study could contribute more than one estimate if different age and sex stratifications or non-overlapping time periods were reported.

### Attributable burden

Schizophrenia was responsible for 0.25% (95% UI 0.21% to 0.29%) and 0.42% (95% UI 0.37% to 0.48%) of total IHD deaths and YLLs respectively, amounting to 22 603 (95% UI 19 475 to 25 998) deaths and 742 715 (95% UI 645 855 to 849 705) YLLs (Table 3). Greater attributable burden was observed in males compared to females, and burden increased with age, peaking at 50 to 54 years (online Supplementary Fig. S2; note that deaths and YLLs showed the same age pattern).

**Table 3.** Estimated burden attributable to schizophrenia

Health outcome	Proportion of deaths, %^a^ (95% UI)	Proportion of YLLs, %^a^ (95% UI)	Attributable deaths (95% UI)	Attributable YLLs (95% UI)
IHD				
Both sexes	0.25 (0.21–0.29)	0.42 (0.37–0.48)	22 603 (19 475–25 998)	742 715 (645 855–849 705)
Female	0.22 (0.18–0.27)	0.44 (0.37–0.51)	9282 (7696–11 110)	304 406 (256 848–356 052)
Male	0.27 (0.21–0.33)	0.41 (0.33–0.49)	13 320 (10 598–16 219)	438 310 (353 239–530 715)
Stroke				
Both sexes	0.20 (0.17–0.23)	0.28 (0.24–0.33)	12 998 (10 914–15 409)	351 820 (295 765–411 293)
Female	0.16 (0.12–0.20)	0.24 (0.19–0.30)	5156 (3923–6545)	134 403 (105 013–168 347)
Male	0.24 (0.18–0.29)	0.31 (0.24–0.39)	7842 (6014–9818)	217 415 (168 908–269 829)
Diabetes				
Both sexes	0.81 (0.77–0.85)	1.08 (1.03–1.13)	12 623 (11 986–13 314)	369 356 (350 940–389 901)
Female	0.92 (0.86–0.98)	1.23 (1.16–1.32)	7317 (6804–7859)	205 387 (191 000–220 766)
Male	0.70 (0.66–0.76)	0.93 (0.87–1.00)	5306 (4927–5738)	163 969 (152 410–176 591)
All-cause				
Both sexes	0.65 (0.59–0.72)	0.82 (0.73–0.91)	368 883 (332 468–408 595)	13 722 580 (12 255 974–15 300 532)
Female	0.53 (0.46–0.62)	0.67 (0.58–0.77)	137 703 (119 024–159 068)	4 816 726 (4 161 168–5 535 587)
Male	0.75 (0.65–0.86)	0.92 (0.79–1.07)	231 180 (199 214–265 150)	8 905 854 (7 657 443–10 251 196)

YLLs, years of life lost; UI, uncertainty interval; IHD, ischaemic heart disease.

a Proportions correspond to attributable burden divided by total burden for each health outcome, converted to percentages.

There were almost half as much stroke deaths and YLLs attributable to schizophrenia compared to IHD; 12 998 (95% UI 10 914 to 15 409) and 351 820 (95% UI 295 765 to 411 293) respectively, which corresponded to 0.20% (95% UI 0.17% to 0.23%) and 0.28% (95% UI 0.24% to 0.33%) of total stroke deaths and YLLs (Table 3). The same sex and age patterns as IHD were observed, with a slightly later peak at age 60 to 64 (online Supplementary Fig. S2).

The largest proportion of disease burden attributable to schizophrenia was for diabetes: 0.81% (95% UI 0.77% to 0.85%) of deaths and 1.08% (95% UI 1.03% to 1.13%) of YLLs (Table 3). This amounted to 12 623 (95% UI 11 986 to 13 314) deaths and 369 356 (95% UI 350 940 to 389 901) YLLs. While the age pattern was the same as IHD and stroke, the sex pattern was reversed, with more attributable burden and proportions of total burden observed in females (online Supplementary Fig. S2).

In terms of all-cause mortality, schizophrenia was responsible for 0.65% (95% UI 0.59% to 0.72%) and 0.82% (95% UI 0.73% to 0.91%) of deaths and YLLs, which corresponded to 368 883 (95% UI 332 468 to 408 595) deaths and 13 722 580 (95% UI 12 255 974 to 15 300 532) YLLs.

IHD, stroke and diabetes together explained 13.08% (95% UI 12.43% to 13.82%) of all deaths and 10.68% (95% UI 10.04% to 11.32%) of all YLLs attributable to schizophrenia, which amounted to a total of 48 223 (95% UI 43 573 to 53 227) deaths and 1 463 891 (95% UI 1 327 260 to 1 607 376) YLLs. This resulted in 320 660 (95% UI 288 299 to 356 517) unexplained deaths and 12 258 690 (95% UI 10 925 426 to 13 713 646) unexplained YLLs (Table 4).

**Table 4.** Unexplained and proportion of explained burden attributable to schizophrenia

	Proportion of explained deaths, % (95% UI)	Proportion of explained YLLs, % (95% UI)	Unexplained deaths^a^ (95% UI)	Unexplained YLLs^a^ (95% UI)
Both sexes	13.08 (12.43–13.82)	10.68 (10.04–11.32)	320 660 (288 299–356 517)	12 258 690 (10 925 426–13 713 646)
Female	15.81 (15.03–16.65)	13.39 (12.59–14.20)	115 947 (996 36 134 437)	4 172 529 (3 580 561–4 821 672)
Male	11.46 (10.61–12.45)	9.22 (8.49–10.09)	204 713 (175 509–235 797)	8 086 160 (6 928 283–9 323 921)

YLLs, years of life lost; UI, uncertainty interval.

a Unexplained burden corresponds to the attributable burden for each health outcome subtracted from all-cause attributable burden.

## Discussion

This is the first study to quantify the physical health burden attributable to schizophrenia as a risk factor. This study goes beyond estimates of elevated mortality risk from previous studies (Correll *et al*., 2022; Lambert *et al*., 2022) to describe specifically how many deaths and YLLs due to cardiometabolic diseases are driven by having schizophrenia. The proportion of attributable burden ranged between 0.16 to 1.23% of the total burden of each health outcome, driven by the low prevalence of schizophrenia which was 0.32% (95% UI 0.27 to 0.37%) globally for both sexes, all ages in 2019 (GBD Collaborative Network, 2020b). For IHD, stroke and diabetes combined, this amounted to around 50 000 deaths and almost 1.5 million YLLs, which is a considerable amount of fatal burden and critically, potentially preventable.

In terms of sex patterns, males had greater attributable burden for IHD and stroke than females, likely due to the overall fatal burden of these diseases being larger in males (GBD Collaborative Network, 2020b). Looking at diabetes mortality in females, the larger RR alongside a higher overall death rate (GBD Collaborative Network, 2020b) resulted in greater attributable burden compared to males. Females also had larger IHD RRs, which is important to consider in light of the greater proportion of CVD deaths noted in the introduction. This may relate to sex and gender-related healthcare disparities, which are apparent for a range of chronic diseases; for example, women are less likely to receive evidence-based treatment for IHD and CVD risk factors including diabetes, which is also a stronger risk factor for vascular disease onset and mortality in women (Prospective Studies Collaboration and Asia Pacific Cohort Studies Collaboration, 2018; Mauvais-Jarvis *et al*., 2020). A study looking at the quality of clinical management of cardiometabolic risk factors in patients with SMD found that women with obesity were less likely than men to receive dietary advice (Ringen *et al*., 2022). Sex differences have also been found in regard to the adverse metabolic risks associated with antipsychotic medication, with greater metabolic disturbances observed in females (Kraal *et al*., 2017). Sex-specific risks and disparities do not appear to be recognised in key recommendations and guidelines for managing cardiometabolic risk factors and physical health conditions in people with SMD (De Hert *et al*., 2009; Galderisi *et al*., 2021; Gronholm *et al*., 2021). Our results warrant this consideration, particularly for the treatment of diabetes and other CVD risk factors in women with schizophrenia.

Around 87% of deaths and 89% of YLLs attributable to schizophrenia could not be explained by the three health outcomes included in the study. This large proportion of unexplained burden points to the contribution of other causes of death. Respiratory diseases are highly prevalent in people with schizophrenia; a recent meta-analysis reported an adjusted prevalence of almost 20% for chronic obstructive pulmonary disease (COPD), the third leading cause of death in the world in 2019 (GBD Collaborative Network, 2020b; Suetani *et al*., 2021). People with schizophrenia are over four times as likely to die of respiratory diseases as defined by ICD-10, which includes both infections and chronic diseases (Ali *et al*., 2022). GBD examines these categories separately, which alongside limited data on more specific causes like COPD, prevented us from including respiratory diseases in the present study. With both the large RR and overall fatal burden, these diseases are likely to explain a significant proportion of the unexplained burden attributable to schizophrenia. While cancer mortality is also consistently elevated, albeit less so with a pooled RR of 1.76, there is a weak or absent link between schizophrenia and cancer incidence (Nordentoft *et al*., 2021; Ali *et al*., 2022). As explored in a recent review, this discrepancy may be due to people with schizophrenia being less likely to receive a cancer diagnosis or effective treatment (Nordentoft *et al*., 2021). The authors also point out the need to investigate mortality from organ-specific cancers due to differing mechanisms and preventative measures, and as with specific respiratory diseases, there is limited data to pool.

Unnatural causes of death may also be responsible for some of the unexplained burden. While natural causes are responsible for the majority of excess deaths in schizophrenia, unnatural causes are associated with the highest mortality risks, with a pooled RR of around 20 for suicide (Ali *et al*., 2022). Even though the amount of underlying burden due to suicide is considerably less than CVD, due to this large RR, there would be a significant proportion of suicide burden attributable to schizophrenia.

This study has several limitations. Firstly, the schizophrenia prevalence data is based on global estimates, which may not correspond exactly with the eight countries included in the analysis. These countries were primarily high-income, which hinders generalisability to LMICs. It is possible that RRs vary by location due to different risk factor profiles and trends in disease burden, however, there is limited data to pool to reliably test for differences (Ali *et al*., 2022). The limited studies also restricted the number of covariates that could be tested and the statistical power to pick up on differences between subgroups. For diabetes, even though there was only one inpatient study, we included population type as a covariate to control for the bias from this study and avoid overestimating the RR, however the magnitude of the covariate should be interpreted with caution. It should also be noted that the single underlying cause of death which the mortality estimates are based on cannot capture the contribution of multiple diseases. This is important to consider in light of people with psychotic disorders being at an increased risk of multimorbidity (Rodrigues *et al*., 2021). Finally, the theoretical framework of the CRA methodology is based on a hierarchical model of causation, which does not take into account the underlying complexity of schizophrenia as a distal risk factor, encompassing a range of interacting causal factors. This extends to the counterfactual risk exposure, the absence of schizophrenia, which is useful for modelling but less applicable to real-world actions.

In terms of future directions, the inherent complexity to this problem calls for further research using approaches designed specifically to address complex causes, such as systems thinking. This methodology can address causes that encompass numerous factors at different levels of influence, while taking into account dynamic and reciprocal relationships (Galea *et al*., 2010). Additionally, more data on specific causes of death in people with schizophrenia is required, in order to create a detailed picture of disease risks, and the targeted preventive measures and treatments that are required.

Highlighting the potentially avoidable disease burden attributable to schizophrenia provides an important evidence base for healthcare planning and practice. In particular, these findings underscore the need for integrated care of mental and physical health. As outlined in the *Lancet Psychiatry* Commission on physical health in people with mental illness, providing holistic care enables the common risk factors, bidirectional interactions and treatments for mental disorders and physical diseases to be addressed together (Firth *et al*., 2019). The authors highlight the need to protect cardiometabolic health from the earliest stages of mental health treatment. Additionally, the substantial amount of attributable burden not accounted for by cardiometabolic diseases underscores the need to quantify other potentially avertable health outcomes for schizophrenia.

In conclusion, this study has produced estimates of the under-recognised burden of schizophrenia as a risk factor for physical health outcomes, providing a means of capturing the excess mortality associated with mental disorders within the GBD framework. The ongoing issue of excess mortality in people with schizophrenia is a matter of health equity and our results demonstrate how much disease burden could be avoided by reducing disparities in physical health, which needs to occur at all stages of the care pathway. Having a mental illness should not be a barrier to leading a healthy life.

## Data Availability

The GBD prevalence and burden data used in this study is publicly available at https://vizhub.healthdata.org/gbd-results/. The data used for the relative risk estimates is available at https://osf.io/tvkfj/.

## Supplementary material

For supplementary material accompanying this paper visit http://doi.org/10.1017/S2045796023000045. click here to view supplementary material
