# Supplementary material for: Schizophrenia as a risk factor for cardiovascular and metabolic health outcomes: a comparative risk assessment
Source: Epidemiol Psychiatr Sci. 2023 Feb 9;32:e8. doi: 10.1017/S2045796023000045 (PMC9971851; doi:10.1017/S2045796023000045)
Supplement: Supplementary file 1 [file S2045796023000045sup001.docx]

# Supplementary material

## Supplementary methods

*Data extraction*

Data was extracted by SA into a Microsoft Excel template and included estimates of excess mortality, uncertainty, the observed and expected number of deaths, age, sex, location, years of start and end of follow-up, sample size, disorder case-definition, sample source, population type, comparison group description, length of follow-up, cause of death definition and adjustment variables (e.g., age, sex, income, comorbidities). Where the age range was not specified, 15–99 was used as this encompassed the majority of age ranges of all-age studies.

*Data analysis*

Where studies reported age-specific and sex-specific estimates without reporting age-sex-specific estimates, the within-study sex ratio was estimated and applied to the age-specific estimates to estimate age-sex-specific estimates with uncertainty around the sex-ratio and age-specific estimates propagated through this process.

Random effects models were used to allow for heterogeneity between and within studies and the multivariate *rma.mv* function was chosen to enable each study to contribute more than one estimate (i.e., different age and sex stratifications or non-overlapping time periods).

In terms of covariates, sex was tested as percent female, the proportion of the sample that was female (centred at 50%), and the age covariate corresponded to the mid-point of the age-range (centred at the mean age, 55). For the two categorical variables, population type and case definition, each level (apart from the reference) was included as a binary covariate. The final model for each health outcome was developed via backward elimination, which involved iteratively removing the least significant covariate until no improvement was seen in the Akaike information criterion using the *fitstats* function.

*All-cause mortality*

For the all-cause meta-regression model, the following covariates were included: age, sex, age-sex interaction, inpatient and mid-year [mid-point of data collection, centred at mean (2000)].

## Supplementary results

A total of 12,646 studies were identified through the systematic review – the PRISMA flow diagram shown in Figure S1 corresponds to the analyses undertaken in (Ali et al., 2022). The 14 studies included in the present analysis correspond to 11 studies that were included in Ali *et al.*’s analyses and 3 studies that were excluded under ‘Other cause-specific’ (as ischemic heart disease, stroke and diabetes were not previously analysed).

Records identified from:

Databases (n = 12,643)

Records removed *before screening*:

Duplicate records removed (n = 2822)

Records screened

(n = 9821)

Records excluded

(n = 9262)

Reports sought for retrieval

(n = 559)

Reports not retrieved

(n = 0)

Reports assessed for eligibility

(n = 559)

Reports excluded:

Wrong diagnosis (n = 101) Wrong comparator (n = 88) Wrong outcomes (n = 75) Comorbidities (n = 54) Restricted population (n = 43) Wrong time period (n = 35) No comparator (n = 34)

Not enough details (n = 23) Repeat cohort (n = 22)

Other cause-specific (n = 5)

Treatment exposure (n = 4) No adjustments (n = 2)

Data request from authors unsuccessful (n = 2)

Records identified from:

Citation searching (n = 3)

Reports assessed for eligibility

(n = 3)

Reports excluded

(n = 0)

Studies included in review

(n = 73)

Reports of included studies

(n = 3)

**Identification of studies via databases and registers**

**Identification of studies via other methods**

**Identification**

**Screening**

**Included**

Reports sought for retrieval

(n = 3)

Reports not retrieved

(n = 0)

Figure S1: PRISMA flow diagram of study selection


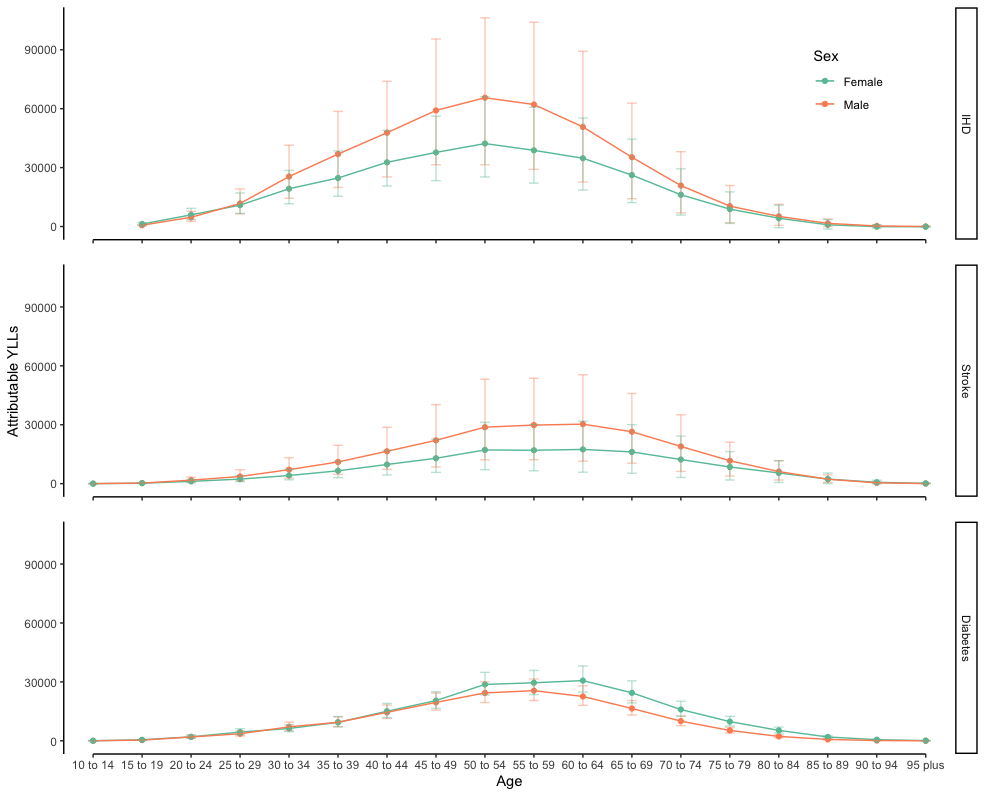


Figure S2: Health outcome YLLs attributable to schizophrenia by age and sex, in 2019. YLLs, years of life lost; IHD, ischemic heart disease. Error bars correspond to 95% uncertainty intervals.

## References

**Ali S, Santomauro D, Ferrari AJ & Charlson F** (2022) Excess mortality in severe mental disorders: A systematic review and meta-regression. *Journal of Psychiatric Research,* 149**,** 97-105.
